# Thermodynamic properties of some isomeric 5-(nitrophenyl)-furyl-2 derivatives

**DOI:** 10.1186/s13065-019-0619-2

**Published:** 2019-08-14

**Authors:** Volodymyr Dibrivnyi, Andriy Marshalek, Iryna Sobechko, Yuriy Horak, Mykola Obushak, Nadiia Velychkivska, Lubomyr Goshko

**Affiliations:** 1 National University “LvivPolytechnic”, S. Bandery Str.,12, Lviv, 79013 Ukraine; 2 Ivan Franko National University of Lviv, Kyryla and Mefodiya Str., 6, Lviv, 79005 Ukraine; 3 Institute of Macromolecular Chemistry, AS CR, Heyrovsky Sq. 2, Prague, Czech Republic; 4 Lviv, Ukraine

**Keywords:** Arylfuran derivatives, Vapor pressure, Combustion enthalpy, Formation enthalpy, Sublimation enthalpy, Isomerization, Group-additivity correlation

## Abstract

**Background:**

The aim of the current work was to determine thermodynamical properties of 5-(nitrophenyl)-2-furaldehyde oximes and 3-[5-(nitrolphenyl)-2-furyl]acrylic acids.

**Results:**

The temperature dependences of saturated vapor pressures of 5-(nitrophenyl)-2-furaldehyde oximes and 3-[5-(nitrolphenyl)-2-furyl]acrylic acids were determined by the Knudsen effusion method. The results are presented by the Clapeyron–Clausius equation in linear form, and via this form, the standard enthalpies of sublimation of compounds were calculated at 298.15 K. The standard molar formation enthalpies of compounds in crystalline state at 298.15 K were determined indirectly from the corresponding standard molar combustion enthalpy, obtained using combustion bomb calorimetry. The non-nearest neighbour interactions (strain) in molecule were defined. The ideal-gas enthalpies of investigated compounds formation and the data available from the literature were used for calculation of group-additivity parameters and the correction terms useful in the application of the Benson correlation.

**Conclusion:**

Determining the thermodynamic properties for these compounds will contribute to solving practical problems pertaining to optimization processes of their synthesis, purification and application. It will also provide a more thorough insight regarding the theoretical knowledge of their nature and are necessary for the application of the Benson group-contribution correlation for calculation of $\Delta {}_{f}H_{{m(298.15{\text{K}})}}^{o}$(g)_calc._

**Electronic supplementary material:**

The online version of this article (10.1186/s13065-019-0619-2) contains supplementary material, which is available to authorized users.

## Introduction

The rapid growth of pharmaceutical and chemical industries using nitrogen-containing heterocyclic compounds requires a continuous diversification of these products. New synthesized compounds with complex structure have no description of their thermodynamic properties. Recently, numerous reactions of synthesis of nitrogen-containing compounds with a phenyl furan fragment, which exhibit various types of biological activity, have been investigated. This allows them to be widely used in various fields of medical chemistry [1–10].

The furfural oximes are used as inhibitors of soil nitrification [1], as intermediates in the synthesis of anti-TB [11] and antifungal [12] drugs, and also as starting materials for the synthesis of disubstituted derivatives of furan [13]. Phenyl derivatives of furfural oxime show antispasmodic [14], vasodilator [15], cardiotropic [8] and antiviral [9] properties.

3-(2-Furyl)acrylic acids are widely used in the synthesis of polymeric materials for the production of polymeric glass, light stabilizers and luminophores [10], as well as for the synthesis of compounds with antimicrobial properties [16].

Previously, we have published several studies on thermodynamic properties of 5-(nitrophenyl)-2-furaldehydes and ethyl esters of cyan acrylic acids [17–19].

This paper follows this course and concerns the investigation of 5-(nitrophenyl)-2-furaldehyde oximes and 3-[5-(nitrolphenyl)-2-furyl] acrylic acids.

The analysis of the properties of positional isomers of disubstituted benzene derivatives shows that a change in the functional group position in an aromatic ring can substantially change the applied properties of compounds, whereas the change in their thermodynamic properties is often unknown. Therefore, the aim of this work is to investigate enthalpy properties of 5-(nitrophenyl)-2-furaldehyde oximes and 3-[5-(nitrolphenyl)-2-furyl]acrylic acids differ in the position of the nitro group.

Investigated 5-(2-nitrophenyl)-2-furaldehyde oxime (A), 5-(3-nitrophenyl)-2-furaldehyde oxime (B), 5-(4-nitrophenyl)-2-furaldehyde oxime (C), 3-[5-(2-nitrolphenyl)-2-furyl]acrylic acid (D), 3-[5-(3-nitrolphenyl)-2-furyl] acrylic acid (E) and 3-[5-(4-nitrolphenyl)-2-furyl] acrylic acid (F) (Table 1) are crystalline substances under normal conditions.

**Table 1:**
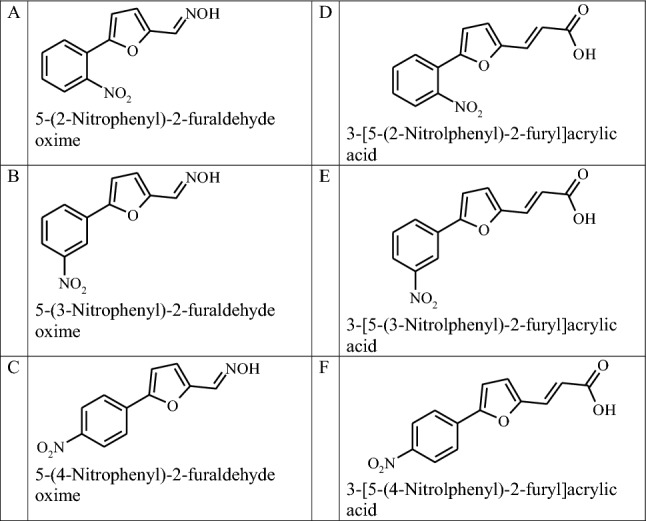
Names and structural formulas of investigated compounds

Thermodynamic properties allow finding the most energetically favourable ways of synthesis and application of compounds with the maximum economic benefit.

Joint analysis of thermodynamic properties of 5-(nitrophenyl)-2-furaldehyde oximes and 3-[5-(nitrolphenyl)-2-furyl] acrylic acids will reveal many theoretically important patterns of mutual influence of atoms in a molecule and enable to calculate the formation enthalpies of free radicals, energy relations, tension, cyclization, determine the group contributions to the additive schemes.

## Results and discussion

### Effusion measurements

Primary effusion measurement results, including the saturated vapor pressure *P* of the researched compounds, are shown in Table 2. The measurement results were processed by the least squares method and presented as a linear equation:

**Table 2:** Results of effusion measurements of investigated substances

T, K	*τ*, s	*m*·10^3^, g	*P*, Pa	*m*·10^3^, g	*P*, Pa	*m*·10^3^, g	*P*, Pa
		Membrane № 1	Membrane № 2	Membrane № 3			
5-(2-Nitrophenyl)-2-furaldehyde oxime							
382.6	7350	1.50	0.097	1.60	0.100	1.50	0.097
386.1	7285	2.45	0.161	2.60	0.165	2.40	0.158
391.4	3655	1.90	0.250	2.00	0.255	2.00	0.264
393.3	3643	2.65	0.350	2.50	0.320	2.50	0.332
396.4	3643	4.05	0.538	4.15	0.534	3.90	0.520
402.1	3660	6.05	0.805	6.55	0.845	6.30	0.842
405.3	3640	10.2	1.37	10.4	1.35	9.70	1.31
5-(3-Nitrophenyl)-2-furaldehyde oxime							
390.0	10,827	2.50	0.111	2.60	0.112	2.60	0.116
394.1	10,828	3.90	0.174	4.00	0.173	3.90	0.174
395.7	10,822	4.70	0.210	4.90	0.212	4.80	0.215
399.4	10,821	6.70	0.301	6.80	0.296	6.70	0.302
402.1	10,825	8.90	0.401	9.00	0.392	8.70	0.393
406.0	7240	8.20	0.555	8.40	0.550	8.10	0.550
410.2	10,830	19.6	0.891	21.0	0.924	20.0	0.912
414.0	10,838	27.5	1.25	28.5	1.26	27.7	1.27
419.8	7222	30.2	2.08	29.9	2.00	29.5	2.04
5-(4-Nitrophenyl)-2-furaldehyde oxime							
414.8	3619	1.75	0.239	1.80	0.238	1.75	0.240
417.6	3619	2.40	0.329	2.50	0.332	2.40	0.330
419.6	3620	2.95	0.406	3.05	0.406	2.95	0.407
421.9	3696	3.75	0.506	3.85	0.504	3.75	0.508
423.1	3622	4.20	0.579	4.25	0.568	4.15	0.575
427.6	3622	6.60	0.915	6.80	0.914	6.55	0.912
429.1	3626	7.70	1.07	7.90	1.06	7.60	1.06
430.2	3600	8.35	1.17	8.55	1.16	8.30	1.17
432.1	3618	10.2	1.42	10.7	1.45	10.3	1.44
3-[5-(2-Nitrolphenyl)-2-furyl]acrylic acid							
422.8	14,418	4.30	0.141	4.40	0.140	4.40	0.145
426.2	7218	3.30	0.217	3.50	0.223	3.30	0.218
426.6	7219	3.55	0.234	3.60	0.229	3.50	0.231
428.9	3620	2.30	0.302	2.40	0.306	2.30	0.304
429.7	7218	5.30	0.350	5.40	0.345	5.30	0.351
430.8	7218	6.20	0.410	6.30	0.404	6.00	0.398
432.2	7220	7.20	0.477	7.60	0.487	7.10	0.472
433.9	3619	4.60	0.609	4.70	0.603	4.50	0.598
434.7	7226	10.0	0.663	10.2	0.656	9.70	0.646
437.2	7223	13.5	0.899	13.9	0.896	13.7	0.915
3-[5-(3-Nitrolphenyl)-2-furyl]acrylic acid							
438.6	10,820	1.55	0.069	1.55	0.067	1.50	0.067
442.3	14,420	3.20	0.107	3.20	0.104	3.25	0.109
444.7	14,428	4.15	0.139	4.35	0.142	4.20	0.142
447.8	14,418	6.10	0.206	5.80	0.190	5.90	0.200
448.4	14,422	6.50	0.219	6.10	0.199	6.00	0.203
449.3	14,416	7.00	0.237	6.90	0.226	6.70	0.227
451.1	10,836	6.40	0.288	6.60	0.288	6.55	0.296
454.7	14,418	11.6	0.394	12.2	0.402	11.0	0.376
456.5	14,417	14.0	0.477	13.9	0.459	13.1	0.448
3-[5-(4-Nitrolphenyl)-2-furyl]acrylic acid							
441.4	21,624	0.65	0.015	0.65	0.014	0.65	0.015
446.2	14,435	0.80	0.027	0.80	0.026	0.80	0.027
448.6	14,400	1.10	0.037	1.20	0.039	1.15	0.039
452.9	14,427	1.80	0.061	1.90	0.062	1.70	0.058
454.2	14,424	1.95	0.066	1.95	0.064	1.95	0.067
455.1	14,422	2.35	0.080	2.35	0.077	2.30	0.079
457.8	14,423	3.10	0.106	3.10	0.102	2.90	0.099
460.2	14,422	3.95	0.135	4.00	0.132	3.85	0.132
463.7	14,422	6.00	0.206	6.30	0.209	6.10	0.210

ln*P* (Pa) = A + B/T with correlation coefficient *ρ*, by means of which the standard molar enthalpies $\Delta_{cr}^{g} H_{m}^{o} \left( {\left\langle T \right\rangle } \right)$ = *B·R* were calculated at average temperatures of measurement interval $\left\langle T \right\rangle$ (Table 3). The error of all experimentally determined thermodynamic values was calculated with the Student’s confidence coefficient of 95%.

**Table 3:** Coefficients of a linear equation: ln P (Pa) = A + B/T. standard sublimation enthalpie of investigated substances

Compound	*T*_*m*_, K	*A*	− *B*, K	*ρ*	$\Delta_{cr}^{g} H_{m}^{o} \left( {\left\langle T \right\rangle } \right)$, kJ mol^−1^
A	394.0	43.2 ± 1.8	17,394 ± 726	0.9934	144.6 ± 6.0
B	404.9	38.83 ± 0.61	15,990 ± 248	0.9988	132.9 ± 2.4
C	423.5	42.9 ± 0.36	18,367 ± 152	0.9996	152.7 ± 1.3
D	430.0	54.38 ± 0.50	23,793 ± 216	0.9992	197.8 ± 2.0
E	447.5	46.3 ± 1.5	21,475 ± 673	0.9951	178.5 ± 5.6
F	452.5	50.2 ± 1.3	24,011 ± 579	0.9971	199.6 ± 4.8

Standard enthalpies of sublimation can be adjusted to 298.15 K by the equations:

1 $$ \Delta_{cr}^{g} H_{m}^{o} \left( {298.15\,{\text{K}}} \right) = \Delta_{cr}^{g} H_{m}^{o} \left( {\left\langle T \right\rangle } \right) + \Delta_{cr}^{g} Cp_{m}^{o} \left( {298.15\,{\text{K}}} \right) \cdot \left( {\left\langle T \right\rangle - 298.15} \right) $$

The changes of standard phase transitions heat capacity values $\Delta_{cr}^{g} Cp_{m}^{o}$ at indicated temperature ranges for the probed compounds are unknown. Therefore, Eq. (2) was used to calculate the enthalpies of sublimation at 298.15 K [20].

2 $$ {{\Delta_{cr}^{g} H_{m}^{o} (298.15\,{\text{K}})} \mathord{\left/ {\vphantom {{\Delta_{cr}^{g} H_{m}^{o} (298.15\,{\text{K}})} {\left( {{\text{kJ}} \cdot {\text{mol}}^{ - 1} } \right)}}} \right. \kern-0pt} {\left( {{\text{kJ}} \cdot {\text{mol}}^{ - 1} } \right)}} = \Delta_{cr}^{g} H_{m}^{o} \left( {\left\langle T \right\rangle } \right) + \left[ {0.75 + 0.15Cp_{{m_{cr} }}^{o} (298.15\,{\text{K}})} \right] \cdot \left( {\left\langle T \right\rangle - 298.15} \right) $$

Heat capacities in solid state $Cp_{{m_{cr} }}^{o}$ were calculated by the additive method [20] and were equal: $Cp_{{m(A,B,C)_{{}} }}^{o}$(298.15 K) = 260.4 J mol^−1^ K^−1^; $Cp_{m(D,E,F)}^{o}$(298.15 K) = 300.7 J mol^−1^ K^−1^. Standard enthalpies of sublimation at the mean experiment temperatures are shown in Table 3.

### Calorimetric measurements

Combustion energy *∆*_*c*_*U(cpd*) of the investigated substances was calculated by the equation:

3 $$ - \Delta_{c} U(cpd) = {{\left( {\Delta U_{\varSigma } - \Delta U_{fuse} - \Delta U_{ter} - \Delta U_{{HNO_{3} }} + \Delta U_{carbon} } \right)} \mathord{\left/ {\vphantom {{\left( {\Delta U_{\varSigma } - \Delta U_{fuse} - \Delta U_{ter} - \Delta U_{{HNO_{3} }} + \Delta U_{carbon} } \right)} {m(cpd)}}} \right. \kern-0pt} {m(cpd)}} $$

where *m(cpd)—*compound weight determined using gas analysis; *ΔU*_*Σ*_ = *W·∆T—*total heat released in the experiment, *W—*energy equivalent of the calorimetric system, *∆T—*real increase in temperature. Calculations were performed taking into account corrections for the combustion of cotton thread *ΔU*_*fuse*_, terylene container *ΔU*_*ter*_, soot to carbon dioxide *ΔU*_*carbon*_ and also for formation in a bomb nitric acid solution $\Delta U_{{HNO_{3} }}$, using the following data for heats of combustion (J g^−1^): terylene 22,944.2 [21]; cotton thread 16704.2 [21]; soot to carbon dioxide − 32,763 [22]; formation of nitric acid 59 kJ mol^−1^ [22].

The results of determination of the compounds combustion energies *∆*_*c*_*U(cpd*) are listed in Table 4, which apart from the above notation, contains values of combustion completeness regarding CO_2_ (the ratio of experimentally determined to calculated weights of CO_2_ *m*_*exp*_/*m*_*cal*_).

**Table 4:** Results of combustion energy determination for investigated compounds

*m(cpd)*, g	*ΔT,* V	*ΔU*_*fuse*_, J	*ΔU*_*HNO3*_, J	*ΔU*_*carbon*_, J	*ΔU*_*ter*_, J	− *∆*_*c*_*U(cpd*), J g^−1^	*m*_*exp*_ */m*_*cal*_
5-(2-Nitrophenyl)-2-furaldehyde oxime							
0.13947	0.25347	106.7	7.7	22.3	412.4	23,464	0.9996
0.18548	0.33140	119.5	11.8	27.6	473.7	23,510	1.0002
0.16006	0.28787	106.2	4.1	20	434.6	23,520	0.9985
0.20085	0.35193	124.4	8.9	39.5	427.2	23,515	0.9961
0.17080	0.30603	118.1	8.9	23.6	441.8	23,507	0.9998
0.17019	0.30680	111.2	9.4	38.2	488	23,510	0.9987
0.19456	0.34651	121.3	11.2	43.5	505.5	23,483	0.9959
− *∆*_*c*_*U(cpd*)_average_ = 23,501 ± 17 J g^−1^							
5-(3-Nitrophenyl)-2-furaldehyde oxime							
0.15358	0.31158	82.0	10.0	28.5	982.4	23,421	0.9968
0.26843	0.46052	94.5	14.8	28.2	499.8	23,400	0.9952
0.14936	0.27347	82.4	7.1	48.4	534.1	23,431	0.9998
0.14361	0.26148	121.2	7.1	25.1	424.5	23,456	1.0000
0.15737	0.28468	120.7	7.1	27.4	453.2	23,438	0.9987
0.18331	0.32799	99.5	8.9	28.2	510.2	23,400	0.9990
0.13068	0.24331	99.4	7.7	25.3	483.8	23,416	0.9991
− *∆*_*c*_*U(cpd*)_average_ = 23,429 ± 16 J g^−1^							
5-(4-Nitrophenyl)-2-furaldehyde oxime							
0.20572	0.36079	106.5	14.2	27.7	486.0	23,319	1.0000
0.12488	0.23304	104.1	4.7	23.9	473.6	23,335	0.9989
0.17515	0.34180	117.1	8.3	32.3	902.4	23,395	0.9999
0.21358	0.40512	120.3	13.0	27.2	946.7	23,335	0.9992
0.18755	0.36144	136.0	9.4	21.3	884.7	23,338	0.9999
0.24203	0.44750	127.9	13.6	31.7	913.2	23,324	0.9987
0.16330	0.32067	120.1	8.9	23.6	858.2	23,360	0.9965
− *∆*_*c*_*U(cpd*)_average_ = 23,344 ± 22 J g^−1^							
3-[5-(2-Nitrolphenyl)-2-furyl]acrylic acid							
0.31440	0.49265	82.0	3.5	29.9	–	23,172	0.9968
0.37839	0.59087	72.3	4.7	28.4	–	23,140	0.9992
0.33991	0.53119	85.7	8.9	53.3	–	23,165	0.9973
0.36358	0.56796	78.7	9.4	30.8	–	23,120	0.9965
0.41155	0.64234	74.8	9.4	30.8	–	23,127	0.9978
0.13428	0.21518	129.3	4.7	34.1	–	23,134	0.9995
0.12360	0.19809	113.3	4.1	28.2	–	23,160	0.9997
− *∆*_*c*_*U(cpd*)_average_ = 23,145 ± 20 J g^−1^							
3-[5-(3-Nitrolphenyl)-2-furyl]acrylic acid							
0.21935	0.3439	97.3	7.1	46.9	–	23,100	0.9985
0.23066	0.36164	103.8	8.9	24.9	–	23,062	0.9962
0.19197	0.30458	110.9	7.7	21.3	–	23,135	1.0000
0.27004	0.42269	80.2	13.0	24.3	–	23,069	0.9998
0.24827	0.39045	97.7	10.6	22.8	–	23,090	0.9986
0.24804	0.38918	84.7	12.4	28.5	–	23,103	0.9991
0.24000	0.37684	91.5	10.0	24.4	–	23,076	0.9979
− *∆*_*c*_*U(cpd*)_average_ = 23,091 ± 22 J g^−1^							
3-[5-(4-Nitrolphenyl)-2-furyl]acrylic acid							
0.16072	0.25432	106.7	5.9	22.8	–	23,020	0.9999
0.17773	0.27984	101.4	7.7	25.1	–	22,989	0.9999
0.23568	0.37014	115.7	10.0	27.2	–	22,985	0.9996
0.16126	0.25492	101.1	5.9	23.3	–	23,036	0.9998
0.19640	0.30968	99.9	8.3	23.0	–	23,062	0.9997
0.16949	0.26797	112.3	6.5	23.5	–	22,997	0.9999
0.16084	0.25462	102.3	4.7	20.3	–	23,050	0.9995
− *∆*_*c*_*U(cpd*)_average_ = 23,020 ± 26 J g^−1^							

The high rate of consistency of carbon dioxide content in substances (0.9952 to 1.0002) calculated by the formula (the results of its experimental determination are shown by the Rossini method) can also serve as an indirect confirmation of the sufficient purity of the compounds.

The absence of significant systematic errors while measuring at the calorimetry installation was confirmed by the coincidence of our results of combustion enthalpies (kJ/mol) of secondary etalons (salicylic acid) and biphenyl − 3026.6 ± 3.1 and − 6246.9 ± 7.3 with recommended ones: − 3025.0 ± 5.0 and − 6250 ± 20 respectively [23].

The standard combustion enthalpies $\Delta {}_{c}H_{m}^{o} (cr)$_(298.15K)_ of compounds were calculated taking into account the correction for the volume expansion work ***∆****nRT* and the Washburn correction [24].

The calculation of formation enthalpies in condensed phase $\Delta {}_{f}H_{{m(298.15{\text{K}})}}^{o}$ by Eq. (4) was based on the following key values of $\Delta {}_{f}H_{{m(298.15{\text{K}})}}^{o}$(kJ mol^−1^): − 285.830 ± 0.042 (H_2_O; l) and − 393.514 ± 0.046 (CO_2_; g) [25].

4 $$ \begin{aligned}   \Delta _{f} H_{{m(298.15K)}}^{o} ({\text{C}}_{{\text{a}}} {\text{H}}_{{\text{b}}} {\text{O}}_{{\text{c}}} {\text{N}}_{{\text{d}}} ({\text{cr}})) = {\text{a}} \cdot \Delta _{f} H_{{m(298.15K)}}^{o} ({\text{CO}}_{{\text{2}}} ({\text{g}})) +  \hfill \\   {\text{b}}/2\Delta _{f} H_{{m(298.15K)}}^{o} ({\text{H}}_{2} {\text{O(1)}}) - \Delta _{c} H_{m}^{o} (cr)({\text{C}}_{{\text{a}}} {\text{H}}_{{\text{b}}} {\text{O}}_{{\text{c}}} {\text{N}}_{{\text{d}}} ({\text{cr}})) \hfill \\  \end{aligned}  $$

The gaseous state standard formation enthalpies of the investigated compounds were determined by the summation of the corresponding solid state formation enthalpies and their sublimation enthalpies according to the Eq. (5).

5 $$ \Delta {}_{f}H_{{m(298.15{\text{K}})}}^{o} \left( {\text{g}} \right) \, = \Delta {}_{f}H_{{m(298.15{\text{K}})}}^{o} \left( {\text{cr}} \right) \, + \Delta_{cr}^{g} H_{m(298.15)}^{o} $$

Standard combustion $- \,\Delta {}_{c}H_{m}^{o} (cr)$ and formation ($- \,\Delta {}_{f}H_{m}^{o} (cr)$,$- \,\Delta {}_{f}H_{m}^{o} (g)$) enthalpies of the investigated compounds are listed in Table 5.

**Table 5:** Combustion and formation enthalpies of investigated compounds at 298.15 K (kJ/mol)

Compound	$- \Delta {}_{c}H_{m}^{o} (cr)$	$- \Delta {}_{f}H_{m}^{o} (cr)$	$\Delta_{cr}^{g} H_{m}^{o}$	$\Delta {}_{f}H_{m}^{o} (g)$		δ
				exp	calc	
A	5458.8 ± 4.0	13.1 ± 4.0	148.4 ± 7.5	135.3 ± 8.5	108.3	27.0
B	5442.0 ± 3.6	29.9 ± 3.6	137.2 ± 3.9	107.3 ± 5.3		− 0.5
C	5422.2 ± 5.2	49.7 ± 5.2	157.7 ± 2.7	108.0 ± 5.9		− 0.3
D	6003.0 ± 5.3	398.7 ± 5.3	203.9 ± 2.3	− 194.8 ± 5.8	− 256.7	61.9
E	5989.0 ± 5.7	412.9 ± 5.7	185.4 ± 6.5	− 227.5 ± 8.6		29.2
F	5970.6 ± 6.8	431.3 ± 6.8	206.7 ± 5.6	− 224.6 ± 8.8		32.1

The standard gaseous state formation enthalpies of compounds $\Delta {}_{f}H_{{m(298.15{\text{K}})}}^{o}$(g)_calc_ are also calculated according to Benson additive scheme [26]. According to the scheme, the formation enthalpy of a compound in the gaseous state is the sum of the contributions of individual groups. Each group consists of a central atom and atoms of its first environment. Structural formulas of substances with indication of the serial number of increment, along with the values of group contributions $\Delta {}_{f}H_{{m(298.15{\text{K}})}}^{o}$(g)_calc_ which were used for calculations of the formation enthalpies are shown in the Tables 6 and 7. Comparison of the experimentally obtained formation enthalpies and those calculated according to the Benson additive scheme allows us to determine the contributions of unknown groups to the additive scheme. In case of experimental and calculated values coincidence, it is possible to calculate these values for other representatives of the investigated compounds. In case of differences, one can find the interaction between non-nearest atoms in a molecule, predict the peculiarities of the compound structure and correct these interactions in Benson additive scheme.

**Table 6:** Calculation of the formation enthalpies of the studied substances using the addictive Benson scheme for 5-nitrophenyl-2-furaldehyde oxime isomers

Substance	№	Increment	*Δ*_*f*_*H*_*298.15*_, kJ mol^−1^
	1–4	C_b_–(C_b_)_2_(H)	13.8
	5	C_b_–(C_b_)_2_(C_d_)	23.8
	6	C_b_–(C_b_)_2_(NO_2_)	− 0.5
	7	C_d_–(C_d_)(C_b_)(O)	59.7
	8,9	C_d_–(C_d_)_2_(H)	28.4
	10	C_d_–(C_d_)_2_(O)	43.4
	11	O–(C_d_)_2_	− 137.2
	12	C_d_–(C_d_)(H)(N–OH)*	33.0
	Furan cycle		− 25.9

Δ_*f*_*H*_*298,15*_ = 4·Δ_*f*_*H*_*298,15*_(C_b_–(C_b_)_2_(H)) + Δ_*f*_*H*_*298,15*_(C_b_−(C_b_)_2_(C_d_)) + Δ_*f*_*H*_*298,15*_(C_b_–(C_b_)_2_(NO_2_)) + Δ_*f*_*H*_*298,15*_(C_d_–(C_d_)(C_b_)(O)) + 2·Δ_*f*_*H*_*298,15*_(C_d_–(C_d_)_2_(H)) + Δ_*f*_*H*_*298,15*_(C_d_–(C_d_)_2_(O)) + Δ_*f*_*H*_*298,15*_(O–(C_d_)_2_) + Δ_*f*_*H*_*298,15*_(C_d_–(C_d_)(H)(N–OH)) + Δ_*f*_*H*_*298,15*_(Furan cycle) = 4·13.8 + 23.8 − 0.5 + 59.7 + 2·28.4 + 43.4 − 137.2 + 33.0 − 25.9 = 108.3 kJ/mol

**Table 7:** Calculation of the formation enthalpies of the studied substances using the addictive Benson schemefor 3-[5-nitrolphenyl-2-furyl]acrylic acid isomers

Substance	№	Increment	*Δ*_*f*_*H*_*298.15*_, kJ mol^−1^
	1–4	C_b_–(C_b_)_2_(H)	13.8
	5	C_b_–(C_b_)_2_(C_d_)	23.8
	6	C_b_–(C_b_)_2_(NO_2_)	− 0.5
	7	C_d_–(C_d_)(C_b_)(O)	59.7
	8,9,12	C_d_–(C_d_)_2_(H)	28.4
	10	C_d_–(C_d_)_2_(O)	43.4
	11	O–(C_d_)_2_	− 137.2
	13	C_d_–(C_d_)(H)(CO)	32.1
	14	CO–(C_d_)(O)	− 140.2
	15	O–(CO)(H)	− 252.3
	Furan cycle		− 25.9

Δ_*f*_*H*_*298,15*_ = 4·Δ_*f*_*H*_*298,15*_(C_b_–(C_b_)_2_(H)) + Δ_*f*_*H*_*298,15*_(C_b_–(C_b_)_2_(C_d_)) + Δ_*f*_*H*_*298,15*_(C_b_–(C_b_)_2_(NO_2_)) + Δ_*f*_*H*_*298,15*_(C_d_–(C_d_)(C_b_)(O)) +3·Δ_*f*_*H*_*298,15*_(C_d_–(C_d_)_2_(H)) + Δ_*f*_*H*_*298,15*_(C_d_–(C_d_)_2_(O)) + Δ_*f*_*H*_*298,15*_(O–(C_d_)_2_) + Δ_*f*_*H*_*298,15*_(C_d_–(C_d_)(H)(CO)) + Δ_*f*_*H*_*298,15*_(CO–(C_d_)(O)) +Δ_*f*_*H*_*298,15*_(O–(CO)(H)) + Δ_*f*_*H*_*298,15*_(Furan cycle) = 4·13.8 + 23.8 − 0.5 + 59.7 + 3·28.4 + 43.4 − 137.2 + 32.1 − 140.2 − 252.3 − 25.9 = 256.7 kJ/mol

The investigated compounds are rather complex, therefore, some group contributions, necessary for the calculations of the formation enthalpies, are absent. Such contributions are marked with asterisks in Tables 6 and 7. They were calculated using reliable gaseous state formation enthalpies of the compounds. The contributions of the groups C_d_–(C_d_)_2_(O), C–(C_b_)_2_(NO_2_) and C_d_–(C_d_)(O)(C_b_) which are absent in the additive scheme were defined from the gaseous state formation enthalpy of vinylfuran [27], nitrobenzene [27] and ethyl-2-cyano-3-(furan-2-yl)-prop-2-enoate [18]. In addition, we had to use the contribution of the whole fragment (C_d_)–CH=N–OH for calculations. This contribution is the sum of contributions of C_d_–(C_d_)(N)(H), N–(C_d_)(OH), and OH–(N). According to the results published in [24], the contribution of N_i_–(OH) group is approximately − 20.9 kJ/mol, but in the following works [28, 29] this group is absent at all. Contributions of the groups C_d_–(C_d_)(N)(H) and N–(C_d_)(OH) in the Benson scheme are also absent. Therefore, the contribution of the fragment (C_d_)–CH=N–OH was calculated from the formation enthalpy of furfural oxime, which we have defined in [30]. It is the optimal choice because the oximes A, B, C can be considered as its derivatives.

The values of the gaseous state standard formation enthalpies of the investigated compounds which were obtained experimentally _*exp*_*Δ*_*f*_*H*_*298.15*_(g) and calculated theoretically _*calc*_*Δ*_*f*_*H*_*298.15*_(g), as well as the difference between them δ are given in Table 5. The resulting difference cannot be explained by the errors of experiments or calculations.

In Ref. [31] interactions between atoms which were by at least two other atoms were called non-nearest neighbour interactions. The result of such interactions caused in general a strain of a molecule. Authors define the strain of a molecule, as the difference between the experimental standard enthalpy of formation $\Delta {}_{f}H_{{m(298.15{\text{K}})}}^{o}$(g)_exp_ and the calculated sum of the strain-free increments $\Delta {}_{f}H_{{m(298.15{\text{K}})}}^{o}$(g)_calc_. A strain of molecules between benzene and furan rings in tert-butylbenzene (9 kJ/mol) is determined [31]. It can exist in the compounds investigated by us. The strain of adjacent nitro and tert-butyl groups (− 25 kJ/mol) is defined in [31].

**Fig. 1:**
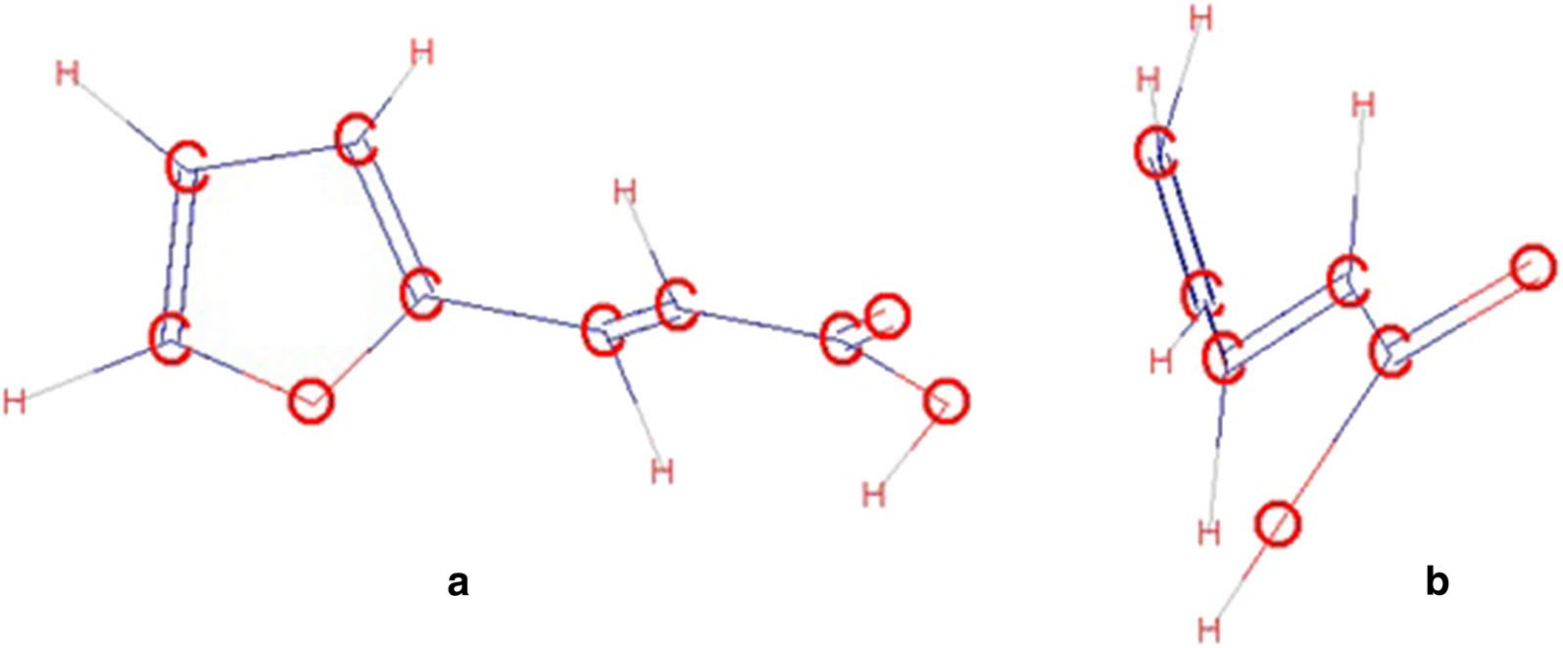
Geometric model of 3-(furan-2-yl)-acrylic acid molecule with minimal internal energy. **a** Frontal projection, **b** side view

Moreover, there is an alternation of single and double conjugated bonds in the molecular structure of all studied compounds. It is known for such systems that the entire molecule is usually situated in the same plane due to the presence of common π-cloud [32]. To verify the assertion, we simulated the most energy-efficient spatial structures of the investigated molecules using the HyperChem software (PM3 geometry optimization method). Other computational calculations of different thermodynamical properties for various nitrogen-containing organical compounds can be found in [33–36].The results confirmed that for the oximes A, B, C the flat configuration of the molecule is the most energy-efficient. However, for the acids D, E, F the minimum internal energy of the molecules is observed when the chain of atoms –CH=CH–C(O)–OH is in a plane almost perpendicular to the furan cycle plane. An example of a geometric model for 3-(furan-2-yl) acrylic acid is shown in Fig. 1. Thus, the strain caused by the furan cycle rotation relative to the chain in the acids D, E and F may exist.

According to the above-mentioned data, we predicted the presence of three different strains in the investigated compounds. The first one is provided by the rotation of the side chain plane relative to the furan cycle in the derivatives of furanacrylic acids (X), the second one corresponds to the interaction of the furan cycle and nitro group in the ortho-position (Y), and the third one is the interaction of the benzene and furan cycles (Z). To calculate interaction corrections in Benson additive scheme a redefined system of six linear equations with two unknowns (X) and (Y) was composed of the corresponding experimentally obtained formation enthalpies and strain-free increments. The correction (Z) of 9 kJ/mol was taken from [31]. The same correction was used to determine the contribution of the group C_d_–(C_d_)(O)(C_B_) from the formation enthalpy of 3-substituted 2-cyano-acrylic acid ethyl ester [18]. As a result of the system solution, the correction terms (kJ/mol) were defined: X = − 30.7, Y = − 29.5 It is clear that the corrections were determined for a small set of substances, and will be defined more exactly as additional experimental material is gained.

## Experimental

### Materials

Furyl-2-oxime (A–C) derivatives were synthesized according to the following procedure. A mixture of appropriated furyl-2-carbaldehyde (0.023 mol), hydroxylamine hydrochloride (0.03 mol) and fused sodium acetate 2 g in 20 mL of ethanol was boiled for 4 h, 30 mL of water was added to the mixture under stirring after cooling. The resulting precipitate was filtered off and recrystallized from ethanol.

Synthesis of 3-[5-(2-nitrolphenyl)-2-furyl] acrylic acids (D–F) was carried out according to the following procedure. 2–3 Drops of piperidine were added to a mixture consisting of appropriated furyl-2-carbaldehyde (0.01 mol) and malonic acid (0.01 mol) in 10 mL of pyridine. The reaction mixture was heated for 2–3 h in a boiling water bath, then cooled, diluted with water (20 mL) and acidified with diluted (1:1) hydrochloric acid. The solution was filtered off, washed with water and dried. The acids were recrystallized from ethanol or mixture of ethanol–DMF solvents. We used the samples obtained after 3- and 4-fold recrystallization.

The identification of substances was confirmed by NMR^1^H spectroscopy data. NMR ^1^H spectra were recorded on Varian 600 (600 MHz) spectrometers in DMSO-*d*_6_ or acetone-*d*_6_. Chemical shifts (δ, ppm) were determined in regards to the signal of DMSO (2.50 ppm). Spectral data for the investigated substances are shown below:

**(A)**—^1^H NMR (600 MHz, Acetone-*d*_6_), δ: 6.96 (d, *J* = 3.5 Hz, 1*H*, furan). 7.41 (d, *J* = 3.5 Hz, 1*H*, furan), 7.47 (s, 1*H*, CH), 7.64 (t, *J* = 8.4 Hz, 1*H*, C_6_H_4_), 7.78 (t, *J* = 8.1, Hz, 1*H*, C_6_H_4_), 7.88 (d, *J* = 8.1 Hz, 1*H*, C_6_H_4_), 7.91 (d, *J* = 7.8 Hz, 1*H*, C_6_H_4_), 11.20 (s, 1*H*, NOH).

**(B)**—^1^H NMR (600 MHz, DMSO-*d*_6_), δ: 7.32 (d, *J* = 3.3 Hz, 1*H*, furan), 7.43 (d, *J* = 3.3 Hz, 1*H*, furan), 7.68 (s, 1*H*, CH), 7.74 (t, *J* = 8.0 Hz, 1*H*, C_6_H_4_), 8.19 (d, *J* = 7.7 Hz, 1*H*, C_6_H_4_), 8.24 (d, *J* = 7.9 Hz, 1*H*, C_6_H_4_), 8.53 (s, 1*H*, C_6_H_4_), 8.05 (s, 1*H*, NOH).

**(C)**—^1^H NMR (600 MHz, DMSO-*d*_6_), δ: 7.36 (d, *J* = 3.6 Hz, 0.1*H*, furan), 7.45 (d, *J* = 3.6 Hz, 1*H*, furan), 7.67 (s, 1*H*, CH), 8.00 (d, *J* = 8.9 Hz, 2H, C_6_H_4_), 8.18 (d, *J* = 8.9 Hz, 2H, C_6_H_4_), 12.10 (s, 1*H*, COOH).

**(D)**—^1^H NMR (600 MHz, DMSO-*d*_6_), δ: 6.17 (d, *J* = 15.8 Hz, 1*H*, CH=), 7.10 (d, *J* = 3.6 Hz, 1*H*, furan), 7.17 (d, *J* = 3.6 Hz, 1*H*, furan), 7.43 (d, *J* = 15.8 Hz, 1*H*, CH=), 7.65 (t, *J* = 7.8 Hz, 1*H*, C_6_H_4_), 7.79 (t, *J* = 7.8 Hz, 1*H*, C_6_H_4_), 7.96 (d, *J* = 7.8 Hz, 1*H*, C_6_H_4_), 8.00 (d, *J* = 7.8 Hz, 1*H*, C_6_H_4_), 12.57 (s, 1*H*, COOH).

**(E)**—^1^H NMR (600 MHz, DMSO-*d*_6_), δ: 7.05 (d, *J* = 3.6 Hz, 1*H*, 3-H furan), 7.05 (d, *J* = 15.8 Hz, 1*H*, CH=), 7.34 (d, *J* = 3.6 Hz, 1*H*, 4-H furan), 7.51 (d, *J* = 15.8 Hz, 1*H*, CH=), 7.63 (t, *J* = 7.9 Hz, 1*H*, C_6_H_4_), 7.78 (d, *J* = 7.7 Hz, 1*H*, C_6_H_4_), 7.90 (d, *J* = 8.0 Hz, 1*H*, C_6_H_4_), 7.95 (s, *J* = 7.8 Hz, 1*H*, C_6_H_4_), 12.06 (s, 1*H*, COOH).

**(F)**—^1^H NMR (600 MHz, DMSO-*d*_6_), δ: 6.49 (d, *J* = 15.8 Hz, 1*H*, CH=), 7.16 (d, *J* = 3.6 Hz, 1*H*, furan), 7.46–7.51 (m, 2H, CH= + furan), 8.13 (d, *J* = 8.9 Hz, 2H, C_6_H_4_), 8.33 (d, *J* = 8.9 Hz, 2H, C_6_H_4_), 12.55 (s, 1*H*, COOH).

The compounds purity was confirmed by a high-performance liquid chromatography using an Agilent 1100 HPLC instrument with a diode matrix and a mass-selective detector on Zorbax SB-C18 column, 4.6 mm × 15 mm; eluent was acetonitrile–water with 0.1% TFA (95:5) under normal conditions. No admixtures in the samples were detected.

### Effusion measurements

Taking into account low volatility of the analyzed substances, the temperature dependences of the saturated vapour pressures were determined by the integral Knudsen effusion method. The design of the apparatus has been adopted from [37]. Construction of the chamber, membranes and experimental procedure was conducted using the recommendations [38].

Three membranes with a diameter of holes № 1—2.050, № 2—2.100 and № 3—2.055 mm were used for effusional research presented in this paper. The membranes are made of nickel foil with a thickness of 0.09 mm.

The vacuum of 0.1 Pa was achieved for 25 ± 15 s. The weight of the effunded substance *m* was determined using analytical scales VLR-20 (± 5·10^−6^ g) as the difference of the effusion camera weight before and after the experiment. The measurement accuracy of the temperature (*T*) and effusion time (*τ*) was ± 0.5 K and ± 1 s, respectively. Effective time (estimated time of effusion in the steady state, in which the weight loss of the effunded substance is equal to that in the transient regime), determined in separate experiments with benzoic acid, equals to 25 ± 5 s and added to the total time of the experiment.

The vapor pressure in the effusion cell *P*_*k*_ was calculated by equation [39]:

6 $$ P_{k} = \frac{m}{KS\tau \alpha }\sqrt {\frac{2\pi RT}{M}} $$

where *τ* is the time of effusion through a hole in the membrane with area *S*; *T*—temperature, *R*—universal gas constant, *M*—molecular weight of the substance, *α*—condensation coefficient.

Investigated nitrophenyl-furyl derivatives are molecular crystals that sublime without a change in their geometry and molecule weight, which allowed us to admit α to be equal to 1 [40].

Clausing coefficient—K, which stands for the membrane’s resistance to molecular flow of vapor for the hole in the membrane, with ratio of length (l) to radius (r) from 0 to 1.5, was determined by the empirical Kennard formula K = 1/[1 + 0.5(l/r)] [41].

The vapor pressure was calculated using correction factor according to the recommendations [42]. For three membranes used in the present work, the factors are equal to 5.12, 5.21 and 5.14 respectively.

Prior to this, the reliability of the effusion installation was checked by benchmark benzoic acid brand K − 1 (the major component content—99.995% mol) by a series of forty experiments.

The dependences of saturated vapor pressure on temperature have the forms:

7 $$ {\text{The membrane No 1}}\,{\text{ln P}} = \left( { 3 3, 3\pm 1, 3} \right) - \left( { 10 5 8 7\pm 4 5 3} \right)* 1/{\text{T}} $$

Δ_sub_H_340,2_ = 88.0 ± 3.8 kJ/mol; ρ = 0.9967.

8 $$ {\text{The membrane No 2}}\,{\text{ln P}} = \left( { 3 3. 1\pm 1. 7} \right) - \left( { 10 5 1 9\pm 5 8 3} \right)* 1/{\text{T}} $$

Δ_sub_H_340,2_ = 87.4 ± 4.8 kJ/mol; ρ = 0.9944.

9 $$ {\text{The membrane No 3}}\,{\text{ln P}} = \left( { 3 3. 1\pm 1. 2} \right) - \left( { 10 5 20 \pm 3 50} \right)* 1/{\text{T}} $$

Δ_sub_H_340,2_ = 87.5 ± 2.9 kJ/mol; ρ = 0.9990.

The resulting dependence of saturated vapour pressure on temperature for three membranes has the form:

10 $$ {\text{ln P}} = \left( { 3 3. 1 8\pm 0. 7 2} \right) - \left( { 10 5 4 2\pm 20 9} \right)* 1/{\text{T}} $$

Δ_sub_H_340,2_ = 87.6 ± 1.7 kJ/mol; ρ = 0.9976.

The average value of the standard enthalpy of sublimation in the temperature range of (332.5–348.0) K was $\Delta_{cr}^{g} H_{m}^{o} (T_{m} )$ = 87.6 ± 1.7 kJ/mol. In order to adjust the standard enthalpy of sublimation to 298 K, from Eq. (1), the standard heat capacity of benzoic acid at 298.15 J/(mol K) in the solid Cp_s_° = 146.76 ± 0.32 [43] and gaseous Cp_g_° = 103.47 [43] state were utilized. Good coincidence of benzoic acid’s sublimation enthalpy adjusted to 298.15 K $\Delta_{cr}^{g} H_{m(298.15)}^{o}$ (kJ/mol) according to the Eq. (1) 91.1 ± 1.8 with the recommended values 89.7 ± 1.0 [43], and 89.0 ± 4.0 [27] shows the absence of significant systematic errors in the effusion installation.

### Calorimetric measurements

The combustion enthalpies of the substances were determined by upgraded calorimeter V-08MA with the isothermal shell.

The temperature in the thermostat was maintained ± 0.03 K. The energy equivalent of the calorimetric systems *W* was estimated by combustion of the reference benzoic acid grade K − 1 (the major component content—99.995% mol, the heat of combustion, taking into account the Jessup factor—26,434.4 J g^−1^) in a series of 12 experiments. The value of *W* was 14,901 ± 11 J V^−1^.

Before combustion beginning the crystalline A, B and C samples were grinded in chalcedony mortar, screened, placed in Terylene ampoules and ignited in the platinum cup. 1 mL of distilled water was added before combustion. The initial pressure of the oxygen, previously purified from the combustible impurities, carbon dioxide and water, was equal to 3.0 MPa. The duration of the initial, main and end periods was—25, 40 and 30 counts, respectively. The initial temperature of the main period in all experiments was 298.15 K. The quantitative analysis of the combustion products for the presence of carbon oxide by the Rossini method [44] with the accuracy of ± 2·10^−4^ g and nitric acid content by titration of the liquid phase in a bomb with a 0.1 M solution of NaOH was carried out after every experiment. The quantities of the carbon dioxide, formed from the combustion of 1 g of Terylene and the cotton thread, were equal to 2.2872 g and 1.6284 g respectively [45]. The anticipated carbon monoxide to be formed during the combustion of products by using detector tubes within ± 5·10^−6^ g, was not encountered. The soot mass was determined by the weighting of the platinum cup before and after combustion with the accuracy of ± 5 × 10^−6^ g. The reliability of gas analyses was controlled by benzoic acid combustion.

Combustion of the investigated compounds is represented by reaction:

11 $$ {\text{C}}_{{\text{A}}} {{\text{H}}}_{{\text{B}}} {{\text{O}}}_{{\text{C}}} {{\text{N}}}_{{{{\text{D}}}({{\text{s}}})}} + ( {{{{{\text{A}}} + {{\text{B}}}} /4}{{{{\text{-C}}}} / 2}}){{\text{ O}}}_{{ 2({{\text{g}}})}} = {{\text{ A CO}}}_{{ 2({{\text{g}}})}} + {{{\text{B}}} / 2}{{\text{ H}}}_{ 2} {{\text{O}}}_{{({{\text{l}}})}} + {{{\text{D}}}/2}{{\text{ N}}}_{{ 2({{\text{g}}})}} $$

A more detailed description of the diffusion unit and combustion calorimeter, experimental procedure and calculations of primary results are presented in [17].

## Conclusions

The determined thermodynamic properties of these compounds will contribute to solving practical problems pertaining to optimization processes of their synthesis, purification and application.

Temperature dependences of vapor pressure have their own practical value for calculation of the parameters for individual stages of the synthesis.

Determining of the thermodynamic properties for these compounds also provides a more thorough insight regarding the theoretical knowledge of their nature.

Using Hyper Chem software (PM3 geometry optimization method) the flat configuration of the phenyl-furan oxime derivatives molecules was established.

Phenyl-furan acid derivatives molecules have the minimum internal energy of the molecule, when the chain of atoms –CH=CH–C(O)–OH is in a plane almost perpendicular to the furan cycle plane.

The comparison of the formation enthalpies of the investigated substances in a gaseous state obtained experimentally with the ones calculated by the Benson scheme allowed to determine the energy of the interaction of a furan ring with a nitro group in the ortho position of the benzene ring (− 30 kJ/mol) as well as the energy of the chain –CH=CH–C(O)–OH rotation in the molecules of phenyl-furan acid derivatives (− 31 kJ/mol).

The new group-additivity parameters and the correction terms for substituted nitrophenyl-furyl derivatives of oximes and acids allowed to applicate the Benson group—contribution correlation to estimate $\Delta {}_{f}H_{{m(298.15{\text{K}})}}^{o}$(g)_calc._ of the compounds has not been investigated yet.

## Additional file

**Additional file 1: Appendix S1.** Cartesian coordinates and computation results for the investigated compounds.

## Data Availability

All of the experimental data is available in the corresponding tables in this article (Additional file 1: Appendix S1).
