# Supplementary material for: Thermodynamic properties of some isomeric 5-(nitrophenyl)-furyl-2 derivatives
Source: BMC Chem. 2019 Aug 14;13(1):105. doi: 10.1186/s13065-019-0619-2 (PMC6694520; doi:10.1186/s13065-019-0619-2)
Supplement: Supplementary file 1 — Additional file 1: Appendix S1. Cartesian coordinates and computation results for the investigated compounds. [file 13065_2019_619_MOESM1_ESM.doc]

**Appendix S1.** Cartesian coordinates and computation results for the investigated compounds.

|  | | | | Energy | | | | kcal/mol | | |
| --- | --- | --- | --- | --- | --- | --- | --- | --- | --- | --- |
|  |  |  |  | Total energy | | | | -67320.3 | | |
|  |  |  |  | Binding energy | | | | | -2733.1 | |
|  |  |  |  | Heat of formation | | | | | 27.8 | |
|  |  |  |  | Electronic energy | | | | | -392028.7 | |
|  |  |  |  | Nuclear energy | | | | | 324708.5 | |
| No atom | X | Y | Z*10^2^ | | No atom | X | Y | | | Z*10^2^ |
| 1 | 3.756 | 2.040 | 6.724 | | 14 | 1.562 | -1.067 | | | 6.724 |
| 2 | 3.705 | 3.139 | 6.724 | | 15 | 1.593 | -2.455 | | | 6.724 |
| 3 | 5.004 | 1.438 | 6.724 | | 16 | 2.487 | -3.077 | | | 6.724 |
| 4 | 5.909 | 2.054 | 6.724 | | 17 | 0.235 | -2.897 | | | 6.724 |
| 5 | 5.103 | 0.049 | 6.724 | | 18 | -0.107 | -3.929 | | | 6.724 |
| 6 | 6.087 | -0.433 | 6.724 | | 19 | -0.544 | -1.753 | | | 6.724 |
| 7 | 3.952 | -0.721 | 6.724 | | 20 | 0.254 | -0.622 | | | 6.724 |
| 8 | 4.025 | -1.820 | 6.724 | | 21 | -1.980 | -1.526 | | | 6.724 |
| 9 | 2.676 | -0.132 | 6.724 | | 22 | -2.340 | -0.483 | | | 6.724 |
| 10 | 2.579 | 1.273 | 6.724 | | 23 | -2.766 | -2.561 | | | 6.724 |
| 11 | 1.277 | 2.014 | 6.724 | | 24 | -4.136 | -2.292 | | | 6.724 |
| 12 | 1.285 | 3.231 | 6.724 | | 25 | -4.496 | -3.172 | | | 6.724 |
| 13 | 0.067 | 1.409 | 6.724 | |  |  |  | | |  |

|  | | | | | Energy | | | kcal/mol | |
| --- | --- | --- | --- | --- | --- | --- | --- | --- | --- |
|  |  |  |  |  | Total energy | | | -67327.4 | |
|  |  |  |  |  | Binding energy | | | -2740.2 | |
|  |  |  |  |  | Heat of formation | | | 20.6 | |
|  |  |  |  |  | Electronic energy | | | -375385.9 | |
|  |  |  |  |  | Nuclear energy | | | 308057.5 | |
| No atom | X | Y | Z*10^2^ | No atom | | X | Y | | Z*10^2^ |
| 1 | 2.577 | 1.014 | 0.236 | 14 | | 1.641 | -1.292 | | 0.236 |
| 2 | 1.551 | 1.428 | 0.236 | 15 | | 1.589 | -2.673 | | 0.236 |
| 3 | 3.675 | 1.873 | 0.236 | 16 | | 2.424 | -3.370 | | 0.236 |
| 4 | 3.466 | 3.358 | 0.236 | 17 | | 0.201 | -3.035 | | 0.236 |
| 5 | 2.335 | 3.803 | 0.236 | 18 | | -0.197 | -4.046 | | 0.236 |
| 6 | 4.431 | 4.096 | 0.236 | 19 | | -0.510 | -1.852 | | 0.236 |
| 7 | 4.973 | 1.351 | 0.236 | 20 | | 0.357 | -0.769 | | 0.236 |
| 8 | 5.848 | 2.017 | 0.236 | 21 | | -1.927 | -1.535 | | 0.236 |
| 9 | 5.159 | -0.026 | 0.236 | 22 | | -2.225 | -0.473 | | 0.236 |
| 10 | 6.176 | -0.435 | 0.236 | 23 | | -2.779 | -2.516 | | 0.236 |
| 11 | 4.068 | -0.885 | 0.236 | 24 | | -4.127 | -2.160 | | 0.236 |
| 12 | 4.227 | -1.971 | 0.236 | 25 | | -4.546 | -3.014 | | 0.236 |
| 13 | 2.765 | -0.371 | 0.236 |  | |  |  | |  |

|  | | | | | Energy | | | kcal/mol |
| --- | --- | --- | --- | --- | --- | --- | --- | --- |
|  |  |  |  |  | Total energy | | | -67327.8 |
|  |  |  |  |  | Binding energy | | | -2740.64 |
|  |  |  |  |  | Heat of formation | | | 20.2 |
|  |  |  |  |  | Electronic energy | | | -370874.8 |
|  |  |  |  |  | Nuclear energy | | | 303547.0 |
| No atom | X | Y | Z*10^2^ | No atom | | X | Y | Z*10^2^ |
| 1 | 2.517 | 0.990 | 0.259 | 14 | | 1.579 | -1.318 | 0.259 |
| 2 | 1.508 | 1.418 | 0.259 | 15 | | 1.532 | -2.700 | 0.259 |
| 3 | 3.611 | 1.844 | 0.259 | 16 | | 2.372 | -3.392 | 0.259 |
| 4 | 3.443 | 2.931 | 0.259 | 17 | | 0.144 | -3.068 | 0.259 |
| 5 | 4.911 | 1.324 | 0.259 | 18 | | -0.247 | -4.082 | 0.259 |
| 6 | 6.091 | 2.245 | 0.259 | 19 | | -0.570 | -1.888 | 0.259 |
| 7 | 5.911 | 3.447 | 0.259 | 20 | | 0.292 | -0.800 | 0.259 |
| 8 | 7.213 | 1.777 | 0.259 | 21 | | -1.989 | -1.579 | 0.259 |
| 9 | 5.100 | -0.063 | 0.259 | 22 | | -2.293 | -0.519 | 0.259 |
| 10 | 6.113 | -0.490 | 0.259 | 23 | | -2.836 | -2.564 | 0.259 |
| 11 | 4.004 | -0.914 | 0.259 | 24 | | -4.186 | -2.215 | 0.259 |
| 12 | 4.163 | -1.999 | 0.259 | 25 | | -4.600 | -3.071 | 0.259 |
| 13 | 2.702 | -0.398 | 0.259 |  | |  |  |  |

|  | | | | | Energy | | | kcal/mol | | |
| --- | --- | --- | --- | --- | --- | --- | --- | --- | --- | --- |
|  |  |  |  |  | Total energy | | | -76211.5 | | |
|  |  |  |  |  | Binding energy | | | -3155.0 | | |
|  |  |  |  |  | Heat of formation | | | -53.7 | | |
|  |  |  |  |  | Electronic energy | | | -461321.1 | | |
|  |  |  |  |  | Nuclear energy | | | 385109.5 | | |
| No atom | X | Y | Z*10^2^ | No atom | | X | Y | | Z*10^2^ | |
| 1 | 3.943 | 1.507 | -2.343 | 15 | | 1.842 | -3.017 | | -2.343 | |
| 2 | 3.877 | 2.606 | -2.343 | 16 | | 2.745 | -3.626 | | -2.343 | |
| 3 | 5.199 | 0.921 | -2.343 | 17 | | 0.492 | -3.478 | | -2.343 | |
| 4 | 6.096 | 1.549 | -2.343 | 18 | | 0.169 | -4.516 | | -2.343 | |
| 5 | 5.316 | -0.467 | -2.343 | 19 | | -0.306 | -2.345 | | -2.343 | |
| 6 | 6.3064 | -0.936 | -2.343 | 20 | | 0.477 | -1.204 | | | -2.343 |
| 7 | 4.176 | -1.252 | -2.343 | 21 | | -1.741 | -2.156 | | | -2.343 |
| 8 | 4.264 | -2.350 | -2.343 | 22 | | -2.096 | -1.113 | | | -2.343 |
| 9 | 2.892 | -0.680 | -2.343 | 23 | | -2.581 | -3.200 | | | -2.343 |
| 10 | 2.777 | 0.724 | -2.343 | 24 | | -2.213 | -4.236 | | | -2.343 |
| 11 | 1.464 | 1.448 | -2.343 | 25 | | -4.050 | -3.071 | | | -2.343 |
| 12 | 1.455 | 2.664 | -2.343 | 26 | | -4.869 | -3.976 | | | -2.343 |
| 13 | 0.4209 | 0.825 | -2.343 | 27 | | -4.568 | -1.819 | | | -2.343 |
| 14 | 1.790 | -1.629 | -2.343 | 28 | | -5.519 | -1.870 | | | -2.343 |

-

|  | | | | | Energy | | | kcal/mol | |
| --- | --- | --- | --- | --- | --- | --- | --- | --- | --- |
|  |  |  |  |  | Total energy | | | -76218.6 | |
|  |  |  |  |  | Binding energy | | | -3162.0 | |
|  |  |  |  |  | Heat of formation | | | -60.7 | |
|  |  |  |  |  | Electronic energy | | | -441864.9 | |
|  |  |  |  |  | Nuclear energy | | | 365646.4 | |
| No atom | X | Y | Z*10^2^ | No atom | | X | Y | | Z*10^2^ |
| 1 | 2.754 | 0.685 | 1.318 | 15 | | 1.866 | -3.028 | | 1.318 |
| 2 | 1.725 | 1.075 | 1.318 | 16 | | 2.721 | -3.700 | | 1.318 |
| 3 | 3.838 | 1.568 | 1.318 | 17 | | 0.490 | -3.426 | | 1.318 |
| 4 | 3.598 | 3.049 | 1.318 | 18 | | 0.122 | -4.450 | | 1.318 |
| 5 | 2.459 | 3.470 | 1.318 | 19 | | -0.257 | -2.264 | | 1.318 |
| 6 | 4.549 | 3.805 | 1.318 | 20 | | 0.582 | -1.158 | | 1.318 |
| 7 | 5.148 | 1.075 | 1.318 | 21 | | -1.678 | -2.002 | | 1.318 |
| 8 | 6.008 | 1.759 | 1.318 | 22 | | -1.984 | -0.945 | | 1.318 |
| 9 | 5.365 | -0.298 | 1.318 | 23 | | -2.573 | -3.000 | | 1.318 |
| 10 | 6.390 | -0.686 | 1.318 | 24 | | -2.262 | -4.054 | | 1.318 |
| 11 | 4.293 | -1.181 | 1.318 | 25 | | -4.033 | -2.789 | | 1.318 |
| 12 | 4.477 | -2.263 | 1.318 | 26 | | -4.902 | -3.645 | | 1.318 |
| 13 | 2.980 | -0.695 | 1.318 | 27 | | -4.478 | -1.509 | | 1.318 |
| 14 | 1.879 | -1.645 | 1.318 | 28 | | -5.431 | -1.505 | | 1.318 |

|  | | | | | Energy | | | kcal/mol | |
| --- | --- | --- | --- | --- | --- | --- | --- | --- | --- |
|  |  |  |  |  | Total energy | | | -76219.1 | |
|  |  |  |  |  | Binding energy | | | -3162.5 | |
|  |  |  |  |  | Heat of formation | | | -61.3 | |
|  |  |  |  |  | Electronic energy | | | -435430.0 | |
|  |  |  |  |  | Nuclear energy | | | 359211.0 | |
| No atom | X | Y | Z*10^2^ | No atom | | X | Y | | Z*10^2^ |
| 1 | 2.820 | 0.778 | 3.796 | 15 | | 1.925 | -2.937 | | 3.796 |
| 2 | 1.800 | 1.182 | 3.796 | 16 | | 2.780 | -3.610 | | 3.796 |
| 3 | 3.892 | 1.659 | 3.796 | 17 | | 0.549 | -3.334 | | 3.796 |
| 4 | 3.698 | 2.741 | 3.796 | 18 | | 0.179 | -4.357 | | 3.796 |
| 5 | 5.204 | 1.170 | 3.796 | 19 | | -0.197 | -2.170 | | 3.796 |
| 6 | 6.366 | 2.120 | 3.796 | 20 | | 0.642 | -1.065 | | 3.796 |
| 7 | 6.151 | 3.317 | 3.796 | 21 | | -1.618 | -1.908 | | 3.796 |
| 8 | 7.494 | 1.680 | 3.796 | 22 | | -1.924 | -0.852 | | 3.796 |
| 9 | 5.428 | -0.212 | 3.796 | 23 | | -2.514 | -2.906 | | 3.796 |
| 10 | 6.451 | -0.615 | 3.796 | 24 | | -2.201 | -3.960 | | 3.796 |
| 11 | 4.35401 | -1.089 | 3.796 | 25 | | -3.974 | -2.697 | | 3.796 |
| 12 | 4.53905 | -2.170 | 3.796 | 26 | | -4.840 | -3.555 | | 3.796 |
| 13 | 3.03991 | -0.605 | 3.796 | 27 | | -4.422 | -1.418 | | 3.796 |
| 14 | 1.93923 | -1.553 | 3.796 | 28 | | -5.375 | -1.417 | | 3.796 |
